# Cartilage graft and temporal muscle fascia graft in revision myringoplasty: a comparison of anatomical and functional results with an innovative surgical technique

**DOI:** 10.3389/fneur.2024.1497162

**Published:** 2025-01-24

**Authors:** Andrea Canale, Marco Boldreghini, Ili Abboud, Patrizia Peluso, Elisa Vestrini, Gluseppe Riva, Andrea Albera

**Affiliations:** Department of Surgical Science, University of Turin, Turin, Italy

**Keywords:** cartilage graft, myringoplasty, temporalis fascia, myringoplasty revision, surgical techniques evaluation

## Abstract

**Background:**

The temporal muscle fascia is the most widely used type of tissue graft in revision myringoplasty procedures. The aim of this study was to verify if the use of a cartilage graft may be a valid alternative to temporalis fascia. Tympanic reperforations are frequent after myringoplasty, especially in complicated, large, or anterior perforations, so we decided to compare the neodrum stability of two different surgical techniques.

**Materials and methods:**

The study was conducted on 42 patients who underwent revision myringoplasty, carried out with the overlay technique, between 2004 and 2020. In all patients, the retroauricular approach was used. The subjects included in the study were divided into 2 groups: the former was treated with a cartilage graft, while in the latter a temporalis fascia graft was used. In the comparison of the two groups, the following outcomes were taken into consideration: engraftment rate, incidence of complications, pre- and postoperative air conduction pure tone average and air-bone gap.

**Results:**

The success rate was 100% for the first group and 83.3% for the second, with a statistically not significant difference. Among the subjects treated with cartilage graft, complications were observed in 8.3% of the cases, while in patients treated with fascia graft the observed complication rate was 43.3% (*p* = 0.03), maybe due to the shorter follow-up period of the first group. The improvement of the air conduction pure tone average was greater with cartilage graft (*p* = 0.02), whereas the difference between air-bone gap closures in the two groups was not statistically significant.

**Conclusion:**

The cartilage graft can represent a valid alternative to temporal muscle fascia, guaranteeing excellent engraftment, fewer complication rate and satisfactory hearing outcomes.

## Introduction

Eardrum perforations are a frequent cause of recurrent otitis media, chronic inflammation of the middle ear, with possible evolution into cholesteatoma and all of its complications. The main goal of myringoplasty is not to improve hearing but to prevent a direct communication between the middle and external ear. This surgery is highly indicated in patients with tympanic perforations suffering from recurrent otitis or patients whom for work reasons or sports, frequently come in contact with water.

Myringoplasty (MPL), also defined as type I Tympanoplasty (TPL) according to the most recent classification (1–4), was introduced by Berthold (5) in 1868 and is currently the most practiced otological surgery (6). The aim of the procedure is the positioning, in the site of the tympanic perforation, of a free graft of connective tissue that acts as a guide for proper re-epithelialization of the mucosa inside, and of the skin outside. In the underlay technique, the graft is placed medial to the tympanic membrane (TM) remnant so that it can heal at the correct level in relation to the annulus and ossicles. Contrarily, in the overlay technique, the epithelial layer is elevated, and the graft is placed lateral to the fibrous layer of the TM remnant and annulus (7). In some cases when there is a favorable anatomy and small enough perforation, the graft can be placed through the perforation, this is known as the Inlay technique. One of the major failures of myringoplasty is the reperforation of the eardrum due to graft rejection, with an infection causing necrosis or atrophy of the TM and subsequent delayed reperforation. Many studies examined several prognostic factors that may influence myringoplasty success rate: the location and the size of perforation, the flogistic condition of the middle ear, the age, the status of the contralateral ear, the surgical techniques (both surgical approach and type of graft) (8). Revision myringoplasty is indicated in case of reperforation of the new eardrum and different grafting materials can be used for the surgery. The temporalis fascia graft was introduced by Heermann in 1958 (9). This kind of tissue has been the common choice for a very long time: it is sufficiently thin, flexible and resistant, it changes shape and aligns itself with the drum. However, it is not always readily available, especially in revision surgery. For this reason, other types of grafts have also been tested as alternatives to temporalis fascia: both heterologous materials, such as bovine pericardium and equine pericardium, and autologous ones (10). Perichondrium, such as tragal perichondrium, is much more resistant than the temporalis fascia but it is not as readily available as the fascia. In many cases, it is used in conjunction with cartilage for grafting. Tragal cartilage alone has also been used for myringoplasty, although less frequently than other options. It adds strength to the eardrum, which makes it extremely useful in case of concomitant retraction pockets. Auricular (conchal) cartilage is also an option, especially in complex or revision cases where additional structural support is essential, although it may lead to more noticeable changes in ear contour, particularly in patients with smaller or more prominent ears. The rigidity of the auricular graft makes it more effective in situations where TM support is critical, such as in large perforations or atelectatic ears. Literature that specifically compares auricular cartilage to tragal cartilage in myringoplasty can be challenging to find, as most studies either examine cartilage versus fascia or focus on cartilage grafts without a direct comparison between specific cartilage types.

The main challenge when using cartilage, considering the reduced flexibility of the graft, is to ensure that all tympanic edges are covered. The literature highlights that cartilage use for revision myringoplasty guarantees both anatomical and functional results comparable to the use of temporal muscle fascia or perichondrium (11–14). In a recent study comparing both grafts, the temporal fascia has been found preferable in terms of duration and auditory results (15). However, cartilage grafts are commonly used in revision surgery, applied with the underlay technique. To our knowledge, no studies have investigated the use of cartilage in the overlay technique yet: the purpose of this study was to evaluate the efficacy and long-term outcome of a cartilage graft applied with the overlay myringoplasty.

## Materials and methods

This is a retrospective study preformed on 42 patients with recurrent tympanic perforation underwent revision MPL. In 34 of these patients, reperforation occurred in the first 6 months after the initial surgery. The revision surgery was performed 12–16 months after the diagnosis of reperforation.

The study was conducted according to the guidelines of the declaration of Helsinki, and approved by the Institutional Review Board (or Ethics Committee) of the Azienda Ospedaliera Universitaria della Scienza di Torino—presidio Molinette, with protocol code: 58243. The surgery was performed with the overlay technique (annular wedge tympanoplasty—AWT) (16), between 2004 and 2020. All surgeries were performed by two surgeons (RA and AC) using similar techniques. Both surgeons did their residency and surgical training under the same university. It’s also worth noting that AC was historically a student of RA.

The overlay technique in our department is chosen whenever there is a perforation of the two anterior quadrants or a perforation bigger than 3 quadrants. In revision myringoplasty for a reperforation with these anatomical characteristics, RA used overlay technique with fascia graft, while AC (author) used cartilage graft. Both surgeons used a retroauricolar approach, under general anesthesia. Autologous grafts (cartilage or muscle temporal fascia) were used in all surgeries.

The patients were divided into two groups: Group 1, with patients who underwent cartilage grafting from the tragus, and Group 2, including patients who underwent temporal muscle fascia graft. In order to harvest the tragal cartilage a cut is made through the skin and cartilage on the medial side of the tragus, preferably leaving 2 mm of cartilage in the dome for aesthetic purposes. The tragal cartilage is harvested together with the pericondrium. The thickness of the cartilage was never modified after harvesting it.

In both groups, the location of the TM perforation, the affected side, the presence of otorrhea at the time of surgery, the average pre- and postoperative hearing thresholds, the success rates and the occurrence of complications were documented.

Hearing assessment was performed by pure-tone audiometry carried out immediately before and 3 months after surgery. According to the American Academy of Otolaryngology guidelines, audiometric results were compared using two variables: air and bone conduction pure-tone average (PTA) at 0.5-1-2-3 kHz frequencies, and the air-bone gap (ABG) closure.

The average time duration of the follow-ups for the two groups were: 8 (± 4.8) years of follow up evaluations in Group 2, and 4.5 (± 2.9) years in Group 1 patients.

Statistical evaluation was performed by applying both the Student’s *t*-test and the 𝜒^2^ test.

The characteristics of the patients and the relative TM perforations are reported in Tables 1, 2.

**Table 1:** Characteristics of the patients.

	Gender		Side		Average age (year)	Bilateral disease	Otorrhea at surgery	Technique
	M	F	R	L				
Group 1—Cartilage graft	1 (8.3%)	11 (91.7%)	4	8	50	4 (33.3%)	5 (41.7%)	Overlay: 100%
Group 2—Temporal muscle fascia graft	13 (43.3%)	17 (56.7%)	16	14	39	11 (36.7%)	6 (20%)	Overlay: 100%

**Table 2:** Distribution of the type of perforation in the two groups.

Perforation	Group 1	Group 2	*p*
Anterior	2 (16.7%)	6 (20%)	1.000
Inferior	1 (8.3%)	4 (13.3%)	1.000
Subtotal	9 (75%)	20 (66.7%)	0.722

## Results

Overall, the analysis of the results did not report a significant difference between revision myringoplasty performed with tragal cartilage graft or temporal muscle fascia graft in terms of anatomical closure of the TM, auditory outcomes, and complication rates.

On the last day of follow-up evaluation, the neotympanic membrane was found to be completely intact in all patients when a cartilage graft was used, while on the contrary we reported a 17.7% rate of reperforation in case of graft with temporal muscle fascia: the difference between groups was not statistically significant (*p* = 0.13).

As regards the other anatomical anomalies of the TM or complications that were found in the postoperative period, which are reported in Table 3, also in this case no statistically significant difference was highlighted between Group 1 (cartilage graft) and Group 2 (temporalis fascia) (*p* > 0.05). As for the overall incidence of at least one post-operative complication, the cartilage graft proved to achieve significantly better results than the temporal muscle fascia graft (*p* < 0.05).

**Table 3:** Anomalies of the TM or complications.

	Cartilage graft (%)	Temporalis fascia (%)	*p*-value (α = 0.05)
Lateralization of the TM	0	13.33	0.18
Retraction of the TM	0	6.67	0.36
Thickening of the TM	8.33	3.33	0.49
Recurrent otitis	0	10	0.25
At least one complication	8.33	43.33	0.03

In order to compare the two groups correctly, the distribution of the type of perforations was studied with the 𝜒^2^ test with Fisher correction (Table 1).

Only complications found on the medium-long term (3 months) were considered.

Table 4 presents the pre and post-operative PTA-AC, PTA-BC and ABG in the two groups.

**Table 4:** Average hearing thresholds and relative standard deviations.

		Pre-operative	Post-operative
PTA AC	Cartilage graft (dB)	59.7 ± 22.4	43.5 ± 25.2
	Temporalis fascia (dB)	46.0 ± 16.5	42.7 ± 22.0
PTA BC	Cartilage graft (dB)	28.2 ± 17.8	23.8 ± 18.9
	Temporalis fascia (dB)	21.2 ± 12.9	23.0 ± 13.0
ABG	Cartilage graft (dB)	31.8 ± 9.4	19.7 ± 12.2
	Temporalis fascia (dB)	24.8 ± 8.4	19.7 ± 14.9

Concerning the auditory results achieved after surgery, a significant improvement of the average pure tone threshold was only obtained in the use of grafts with cartilage, while there was a fair but not significant improvement in the case the myringoplasty was performed with temporal muscle fascia. None of the patients demonstrated significant decline in BC thresholds 3 months after surgery (*p* > 0.05) and the postoperative ABG resulted similar between groups (*p* > 0.05).

Pre and post-operative data of the two groups were compared (Table 5).

**Table 5:** Comparison of pre and post-operative results.

	Group 1 (dB)	Group 2 (dB)	*p*-value (α = 0.05)
Δ AC Pre-post	16.46 (DS = 16.23)	3.33 (DS = 15.72)	0.02
Δ BC Pre-post	4.38 (DS = 9.18)	-1.78 (DS = 4.52)	0.005
Δ ABG Pre-post	12.08 (DS = 9.75)	5.12 (DS = 14.4)	0.13

It was found that patients operated with a cartilage graft had significantly greater improvements in the thresholds for air conduction than those operated with a fascia graft. This was also demonstrated in other studies (17).

### Discussion

Opinions about the actual benefit of revision myringoplasty are controversial and the limited available literature reporting its results is sometimes used to support this contention. However, different studies show that revision surgery has a success rate above 90% (18). As other studies stating that cartilage graft is the preferable choice in many cases (19, 20), the percentages of engraftment we obtained were 100% for the cartilage graft and 83.33% for the fascia graft. Although the difference between the two groups was not statistically significant, the literature (21) reports success rates of 93.8–98% for cartilage grafts, 80.2–80% for the temporalis fascia grafts: these values are not dissimilar to those obtained in our study.

The results obtained in the two groups of this study are comparable because the distribution of the type of perforations is homogeneous—the perforation of 2 or more quadrants of the MT was considered. The side and size of the perforations are two of the most significant factors for the success rate of revision myringoplasty. For this reason, two considerations can be made: subtotal lesions usually have a lower success rate than smaller ones (22), and anterior perforations are more difficult to repair because of the reduced vascular supply, relatively poor visualization and lack of support (23). The aim of this study is to verify if the use of a cartilage graft may be a more suitable alternative to temporal muscle fascia grafts in revision myringoplasty. A statistically significant difference (*p* = 0.03) was observed in the percentages of patients with late complications: 8.33% of the patients treated with cartilage graft developed at least one late complication, compared to 43.33% of those treated with fascia.

The improvement of the bone thresholds of the patients in the post-operative period is justified by a variation of the resonance, as evidenced also by Stenfelt’s studies, which show that bone conduction thresholds, to a lesser extent than the AC, are also affected by the middle ear.

A thickened TM was found in one patient in Group 1 (8.33%). As for Group 2, five patients presented with a perforation of the neo-eardrum (16.67%); similar results were also encountered in other studies (24, 25).

In four patients we observed lateralization (13.3%), in two patients retraction pockets had formed (6.67%), and one patient presented with a thickened neo-eardrum (3.33%). In three patients, we observed a recurrence of otitis (10%). These data are reported in Table 3.

Thickening of the graft (8.33%) was the only complication observed in the cartilage group, which was expected considering the natural properties of the material. Frequent complications of revision MPL such as lateralization, retraction or re-perforation, found in those treated with fascia, were not observed in the cartilage graft group. This could be explained by the naturally high resistance and stability of the cartilage when compared to fascia. There was also a significantly greater improvement in the air conduction thresholds (*p* = 0.02) in the group treated with cartilage. For reasons related to the material itself, re-epithelialization is slower in the cartilage graft, exposing the neo-eardrum to a higher risk of developing myringitis. Other scientific studies used the incidence of different post-operative complications, such as tinnitus or taste disturbance, and for this reason are not comparable to this evaluation.

The main limitation of this study is the low sample size, which may not represent the results of the clinical practice (100% success rate), even if the prospects are positive. The difference in the time duration of the follow-ups for the two groups (4.5 ± 2.9 years for Group 1; 11 ± 4.8 years for Group 2) could also be considered a limit, even if the increased risk of developing complications (especially reperforations) occurs within the first year after surgery. Group 1 has a follow-up of only 4.5 years on average because the use of the overlay cartilage graft is an innovative technique that has only been tested for about 5 years. Another limitation of the study is its retrospective nature.

### Conclusion

To date, temporalis muscle fascia is used much more frequently in revision MPL, while cartilage is rarely used. A classic example where cartilage grafting is indicated is after repeated perforations, when a definitive closure is sought. In our study, the new cartilage graft overlay technique showed promising results, achieving a 100% successful engraftment rate and good functional auditory results (postoperative PTA-ABG < 20). It also demonstrated a lower incidence of post-operative complications compared to the group treated with temporal muscle fascia (partially attributable to the shorter duration of follow-up in Group 1 compared to Group 2). Cartilage grafting is a valid alternative to temporalis fascia for MPL revision surgery, as the stability of this graft offers the possibility of long-term TM integrity, without sacrificing auditory functions.

## Data Availability

The raw data supporting the conclusions of this article will be made available by the authors, without undue reservation.
